# Accelerated age-related decline in hippocampal neurogenesis in mice with noise-induced hearing loss is associated with hippocampal microglial degeneration

**DOI:** 10.18632/aging.103898

**Published:** 2020-10-11

**Authors:** Hong Zhuang, Jing Yang, Zhihui Huang, Haiqing Liu, Xiaobo Li, Hongyu Zhang, Jiadong Wang, Shen Yu, Kefei Liu, Rui Liu, Mingze Bi, Jian Wang, Richard J. Salvi, Bohua Hu, Gaojun Teng, Lijie Liu

**Affiliations:** 1 Department of Physiology, Medical College, Southeast University, Nanjing 210009, China; 2 Institute of Life Sciences, Southeast University, Nanjing 210096, China; 3 Medical College, Southeast University, Nanjing 210009, China; 4 Kangda College of Nanjing Medical University, Lianyungang 222000, China; 5 School of Human Communication Disorder, Dalhousie University, Halifax, Nova Scotia B3H 4R2, Canada; 6 Center for Hearing and Deafness, University at Buffalo, The State University of New York, Buffalo, NY 14214, USA; 7 Jiangsu Key Laboratory of Molecular Imaging and Functional Imaging, Department of Radiology, Zhongda Hospital, Medical School, Southeast University, Nanjing 210009, China

**Keywords:** noise-induced hearing loss (NIHL), aging, hippocampal neurogenesis, microglial morphology, microglial dystrophy

## Abstract

Large-scale epidemiological surveys suggest that hearing loss (HL) is a significant risk factor for dementia. We previously showed that noise-induced HL (NIHL) impairs hippocampal cognitive function and decreases hippocampal neurogenesis and neuronal complexity, suggesting a causal role of HL in dementia. To further investigate the influence of acquired peripheral HL on hippocampal neurogenesis with the aging process as well as the underlying mechanism, we produced NIHL in male CBA/J mice and assessed hippocampal neurogenesis and microglial morphology in the auditory brain and hippocampus at 4 days post-noise exposure (DPN) or 1, 3, 6, or 12 months post-noise exposure (MPN) by immunofluorescence labeling. We found that the age-related decline in hippocampal neurogenesis was accelerated in mice with NIHL. Furthermore, in mice with NIHL, prolonged microglial activation occurred from 1 MPN to 12 MPN across multiple auditory nuclei, while aggravated microglial deterioration occurred in the hippocampus and correlated with the age-related decline in hippocampal neurogenesis. These results suggest that acquired peripheral HL accelerates the age-related decline in hippocampal neurogenesis and that hippocampal microglial degeneration may contribute to the development of neurodegeneration following acquired peripheral HL.

## INTRODUCTION

Hearing loss (HL), one of the most frequent sensory deficits in humans, is the fourth highest cause of disability globally [1]. Growing epidemiologic and clinical evidence has linked peripheral HL with accelerated cognitive decline and identified it as a risk factor for all-cause dementia [2–5]. The Lancet Commission on Dementia Prevention, Intervention, and Care suggested that 35% of dementia cases are attributable to nine potentially modifiable health and lifestyle factors, among which HL was ranked as the largest risk factor for dementia risk from midlife to late life [6].

Noise is the most significant and preventable cause of acquired peripheral HL [7]. With the rapid urbanization and industrialization of modern society, noise exposure can occur in transport, industrial and recreational activities and is pervasive in everyday life. Considering the abundant opportunities for noise overexposure and the enormous global challenge faced in the prevention of dementia due to its rising prevalence and the lack of an effective cure, it is important to scrutinize the nature and underlying mechanism of the relationship between cognitive decline and HL in animals with NIHL.

In our previous studies, CBA mice with NIHL exhibited a marked decline in cognitive function on the Morris water maze (MWM) task at 3 months post-exposure, suggesting that NIHL could be a causal factor in cognitive impairment [8, 9]. Furthermore, we found that cognitive decline in mice with NIHL was associated with impaired adult hippocampal neurogenesis [8, 9]. Because hippocampal neurogenesis has been linked to learning and memory [10], the NIHL-induced reduction in hippocampal neurogenesis could be an important factor linking HL to cognitive decline [8, 9]; however, the biological mechanisms by which peripheral NIHL impairs neurogenesis and cognitive decline are poorly understood.

Acquired HL resulting from cochlear damage caused by mechanical lesions or intense noise can reduce the neural output from the cochlea to the central nervous system (auditory deprivation), initiate a complex series of activity-dependent changes in the auditory brain, and result in long-term neuroplastic changes and neural reorganization [11–14]. Studies performed using different animal models, including rat [12, 13, 15, 16], have reported persistent microglial activation in the cochlear nucleus after auditory deprivation caused by cochlear damage.

Microglia, the predominant resident immune cells of the CNS, are ubiquitously distributed in the brain and integrate with the neural tissue via distinct ramified processes through which they constantly survey the surrounding neuronal network [17–20]. Under homeostatic conditions in the healthy brain, surveilling microglia display a ramified phenotype, marked by a small soma and numerous long, thin processes with delicate arborization (also called ramified microglia). In response to infection or injury, microglia can be activated and assume an amoeboid-like phenotype, characterized by shorter, thicker processes and a larger soma, with increased expression of phagocytic markers, such as CD68 [20, 21]. The traditional role of microglia has been in host defense, such as phagocytosing debris and aberrant proteins as well as secreting factors to promote healing and tissue repair. Recent evidence extends their role to homeostasis of the healthy brain, including processes related to neuroplasticity, such as synaptic stripping [16, 22–24] and neurogenesis [25–27]. In the hippocampus, microglia are very abundant and have been identified as an essential component of the neurogenic niche in the subgranular zone (SGZ) [28] that functions as a key neuromodulator in neurogenesis [28–30]. Ramified microglia were found to play a critical beneficial role in maintenance of the neurogenic cascade via the removal of apoptotic newborn cells and the production of neurotrophic factors [26, 27] and are recognized as versatile modulators of neurogenesis [26, 27, 31]. Degenerative microglia, characterized by dystrophic morphological changes, including significant reductions in soma size and territory area, as well as deramification and process fragmentation, are believed to lose their beneficial neuroprotective functions [21, 32–34] and have been reported to be causally related to the development of suppressed neurogenesis [35]. In addition, multiple studies have demonstrated the deleterious effect of microglial activation on neurogenesis [36, 37]. Thus, appropriate microglial function is essential for neurogenesis.

Microglia are rigorously regulated by factors within the CNS microenvironment and can rapidly respond to extrinsic cues in their environment by transitioning from a surveillance mode to distinct phenotypes, culminating in either beneficial or detrimental outcomes [19, 38]. Thus, microglia are considered the most susceptible sensors of brain pathology [20] and have been proposed to serve as a functional hub that integrates information from inner and outer brain regions and regulates neurogenesis by distributing output information to modulate neuronal plasticity [30, 39]. While microglial activation in the cochlear nucleus after HL was thought to play a pivotal role in neuronal circuit protection and reorganization [12, 13, 15, 16], it is currently unclear whether NIHL leads to microglial alterations in higher parts of the auditory brain or hippocampus. Since strong anatomical and functional connections exist between auditory and cognitive brain regions [40] and acquired peripheral HL impacts hippocampal neurogenesis [8, 9], we speculated that microglial alterations in the hippocampus would be involved in the development of neurogenesis deterioration following acquired HL. To test this hypothesis, we established an NIHL model using CBA mice that were exposed to white noise at a sound pressure level (SPL) of 120 dB for 2 h and searched for evidence of microglial alterations at sites along the ascending auditory pathway and in the hippocampus. Then, we investigated whether changes in adult hippocampal neurogenesis correlate with microglial alterations in the hippocampus.

## RESULTS

### Permanent NIHL

**Figure 1:**
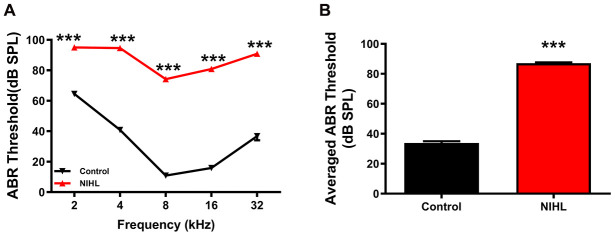
**A significant elevation in the ABR threshold was induced by noise exposure in the NIHL group.** (**A**) ABR frequency-threshold curves for the NIHL and control groups. (**B**) ABR frequency-averaged thresholds for the NIHL and control groups. The values are presented as the mean ± SE of 32 mice per group. *** P<0.001 in *post hoc* comparisons between the NIHL group and the control group using two-way repeated measures ANOVA (**A**) or *t*-test (**B**).

The auditory brainstem response (ABR) frequency-threshold curve obtained at 3 weeks post-exposure was significantly higher in NIHL mice than in control mice at all frequencies, ranging from 2-32 kHz (Figure 1A, two-way repeated measures ANOVA (F_1, 89_ =2264.37, Tukey’s post hoc test P<0.001)). The mean threshold of the NIHL group was significantly higher than that of the control group (Figure 1B, 87.08±0.5702 dB SPL vs. 33.82±1.159 dB SPL, P<0.0001).

### Age-related decline in hippocampal neurogenesis is accelerated by NIHL

**Figure 2:**
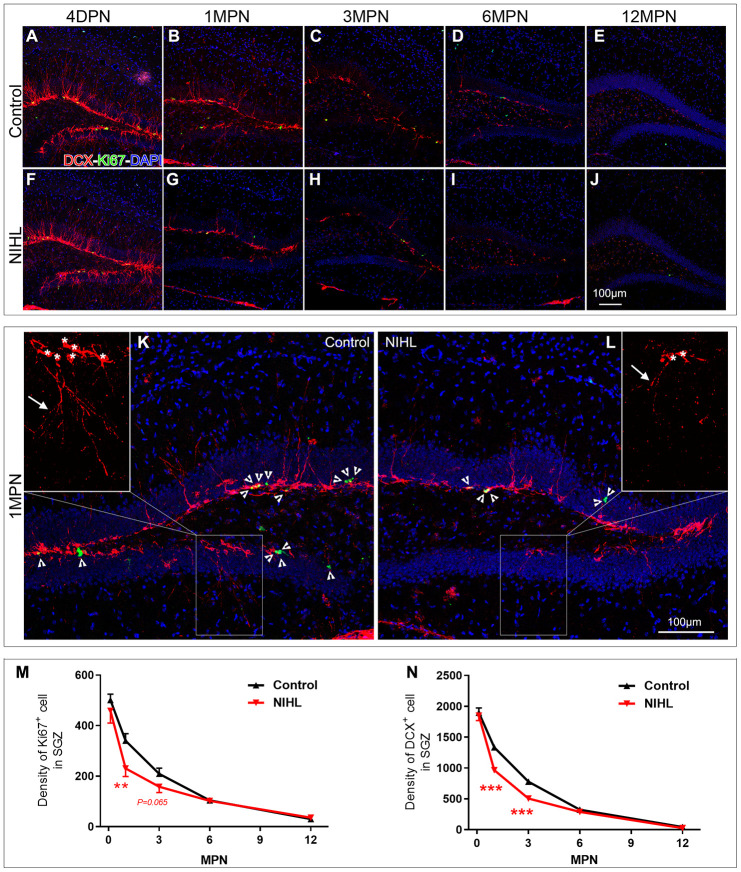
**NIHL mice exhibited an accelerated age-related decline in hippocampal neurogenesis.** (**A**–**J**) Representative images of Ki67^+^ (green) and DCX^+^ (red) cells in the hippocampal DG of control and NIHL mice at 4 DPN (**A**, **F**), 1 MPN (**B**, **G**), 3 MPN (**C**, **H**), 6 MPN (**D**, **I**) and 12 MPN (**E**, **J**). Note the accumulation of autofluorescent lipofuscin deposits (red), a sign of normal senescence [43], in the DG of control and NIHL mice at 6 MPN and 12 MPN. Scale bar: 100 μm. (**K**, **L**) Enlarged views of B (**K**, flipped horizontally) and G (**L**). Arrowheads indicate Ki67^+^ (green) cells. Asterisks and arrows in the magnified inserts indicate the soma and processes of DCX^+^ cells (red). Note the obvious reduction in branch complexity of DCX^+^ cells in NIHL mice. Scale bar: 100 μm. (**M**, **N**) Quantitative analyses of Ki67^+^ cells (**M**) and DCX^+^ cells (**N**) in the DG of mice at different time points. **P<0.01, ***P<0.001 (two-way ANOVA, *post hoc* Tukey’s test, vs. age-matched control group).

We previously demonstrated that NIHL depressed hippocampal neurogenesis at 3 MPN using endogenous (Ki67 and DCX) or exogenous (BrdU and Edu) neurogenesis markers [8, 9]. We extended our findings by examining the effects of NIHL on hippocampal neurogenesis by assessing neurogenesis with Ki67 and DCX at 4 DPN, 1 MPN, 3 MPN, 6 MPN, and 12 MPN (animal ages of ~3, 5, 8 and 14 months, respectively). Figure 2A–2L shows representative images of Ki67 and DCX immunostaining in the DG from mice sacrificed at each designated time point. Quantitative analyses of Ki67^+^ cells and DCX^+^ cells in the DG of mice at different time points are presented in Figure 2M–2N.

Ki67^+^ (Figure 2M) and DCX^+^ (Figure 2N) cells were observed in the DG of the NIHL and control groups at each time point; however, the density of labeled cells declined with age and varied across treatment groups. The densities of Ki67^+^ cells and DCX^+^ cells decreased from 4 DPN to 12 MPN, clearly demonstrating a negative effect of advancing age on neurogenesis [41]. In the control group, using the value from 4 DPN mice (approximately 2 months old) as 100%, the densities of Ki67^+^ cells and DCX^+^ cells decreased from ~30% (32.2% and 29.5%, respectively) at 1 MPN (3 months old) to ~58% (58.2% and 59.0%, respectively) at 3 MPN (5 months old). In the NIHL group, similar but faster declines in Ki67^+^ cell density and DCX^+^ cell density occurred as a function of age (Figure 2M, 2N). Compared with the corresponding value at 4 DPN, the densities of both Ki67^+^ cells and DCX^+^ cells decreased ~48% (49.6% and 47.8%, respectively) at 1 MPN and ~68% (65.5% and 72.7%, respectively) at 3 MPN.

Two-way ANOVA for the factors hearing (NIHL vs. control) and age (time point) revealed significant effects of both NIHL and aging on the density of Ki67^+^ (NIHL: F_1,48_ =6.708, P=0.013; age: F_4,48_ =137.478, P<0.001) and DCX^+^ cells (NIHL: F_1,48_ =42.756, P<0.001; age: F_4,48_ =918.505, P<0.001). A significant NIHL-by-age interaction was observed for the density of DCX^+^ cells (F_4,48_ =6.877, P<0.001) but not Ki67^+^ cells (F_4,48_ =1.993, P=0.111). Although the densities of both Ki67^+^ cells and DCX^+^ cells were comparable between the two groups at 4 DPN and after 6 MPN, they were significantly lower in NIHL groups at 1 MPN, whereas at 3 MPN, there was a significant difference between the NIHL and control groups for only DCX^+^ cells.

### Microglia in the auditory brain regions of NIHL mice assume an activated (hypertrophic) morphology

**Figure 3:**
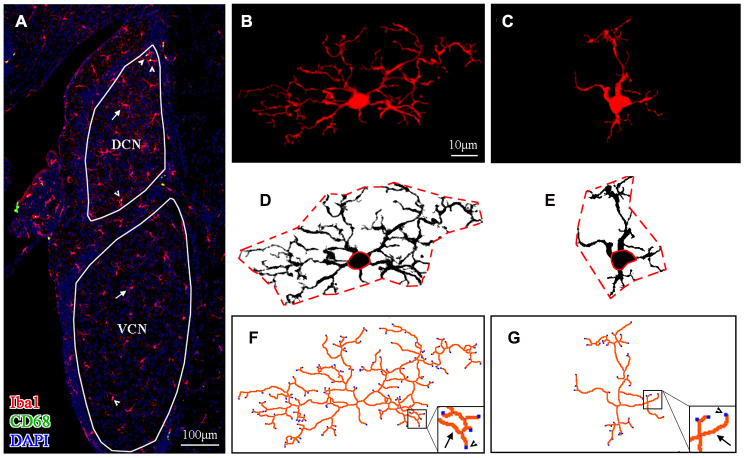
**Morphological analysis of microglia.** (**A**) A representative z-projection image of Iba1- and CD68-labeled sections showing the distribution of microglia in the brain region of interest (the VCN and DCN were delineated in this image). The long arrows point to the cells that were Iba1-positive only, and the short arrows point to cells that were both Iba1- and CD68-positive. The scale bar equals 100 μm. (**B**, **C**) Representative maximum intensity projections of confocal images of an Iba1-labeled ramified microglial cell (**B**) and an Iba1-labeled activated microglial cell (**C**). (**D**, **E**) The soma area and the territory area of microglia (in **B** and **C**) were delineated by a solid (soma area) red line and a dotted (territory area) red line (in **D** and **E**), respectively. (**F**, **G**) Maximum intensity projections of confocal images (**B** and **C**) were skeletonized. The total process length (orange lines, indicated by arrows in the enlarged insert) and the total number of process endpoints (blue spots, indicated by arrowheads in the enlarged insert) were summarized for statistical comparisons. The scale bar equals 10 μm (**B**–**G**).

Figure 3A shows the distribution of microglial cells in the auditory brain regions of interest, with the ventral cochlear nucleus (VCN) and dorsal cochlear nucleus (DCN) serving as examples. Representative maximum intensity projections of confocal images of an Iba1-labeled ramified microglial cell and an Iba1-labeled activated microglial cell are shown in Figure 3B and 3C, respectively. For morphological analysis, the maximum intensity projection of Iba1-positive fluorescence was converted to a binarized format (Figure 3D, 3E) and then skeletonized (Figure 3F–3G) using ImageJ software. Data on the soma area (delineated by the solid red line in Figure 3D, 3E), territory area (delineated by the dotted red line in Figure 3D, 3E), total process length (orange lines in Figure 3F, 3G), and total number of process endpoints (blue spot in Figure 3F, 3G) for individual microglia were assessed.

**Figure 4:**
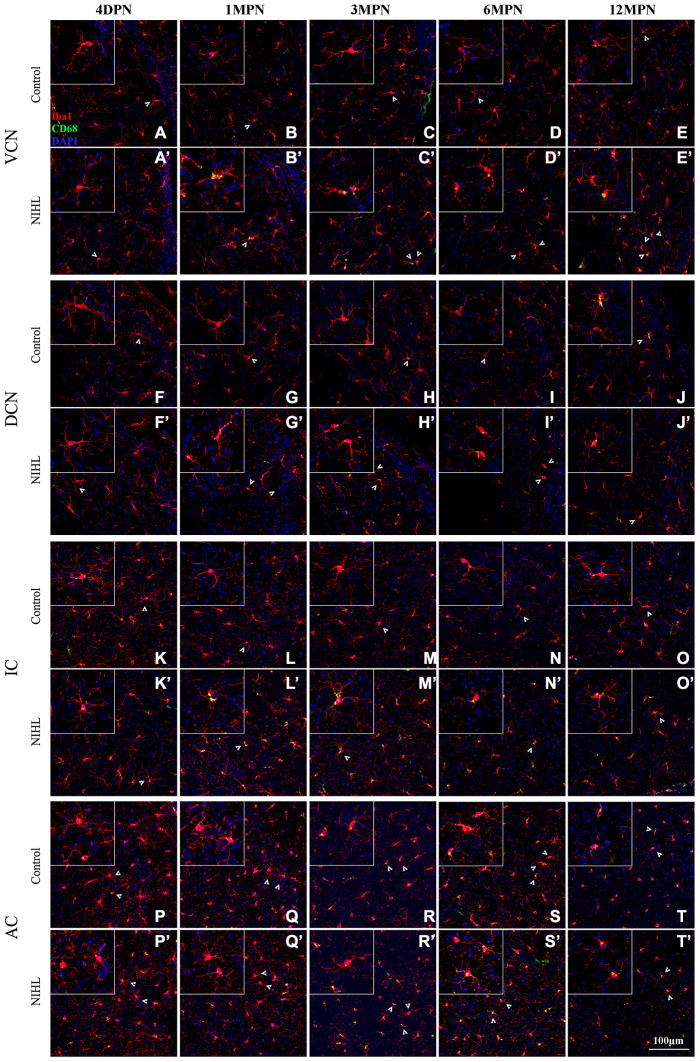
**Microglia in the auditory brain regions of NIHL mice assumed an activated morphology.** (**A**–**T**) Representative z-projection images of Iba1-, CD68-, and DAPI-labeled control brain sections from the VCN (**A**–**E**), DCN (**F**–**J**), IC (**K**–**O**), and AC (**P**–**T**). (**A’**–**T’**) Representative images of corresponding brain sections from NIHL mice. The insets show higher magnifications of the corresponding Iba1^+^ cells signified by arrowheads. The scale bar equals 100 μm.

Figure 4 shows representative confocal images of microglial cells stained for Iba-1 and CD68 from each group across different regions and time points. Similar to previous reports [21, 32, 42], the microglia exhibited heterogeneous morphologies in terms of cell density and process ramification in a region- and age-dependent manner. In the control mice, microglial cells in the VCN, DCN, inferior colliculus (IC) and auditory cortex (AC) displayed slight alterations in morphology with aging, ranging from the very delicate arborization of processes at 4 DPN (Figure 4A, 4F, 4K, and 4P) to shorter processes with fewer branches and even abnormally twisted processes at 12 MPN (Figure 4E, 4J, 4O, and 4T), accompanied by a slight increase in CD68 occupancy, accompanied by slightly increased CD68 occupancy, representing an aging phenotype of microglia [32, 43, 44].

Overall, NIHL increased the microglial density (Figure 4C’–4D’) and resulted in increased microglial activation in auditory nuclei, as reflected by increased CD68 occupancy (Figure 4B’–4E’, 4H’–4I’, 4L’–4N’), enlarged cell soma (Figure 4B’, L’), decreased territory area (Figure 4B’–4D’, 4N’), and shorter but robust (Figure 4C’, 4I’, 4N’) or deramified processes (Figure 4C’, 4D’, 4N’) [45–47].

Table 1 summarizes the results of two-way ANOVA for the effects of the factors hearing (NIHL vs. control) and age (time point) on all six measurements of microglial status. As summarized in Table 1, the analysis revealed not only a significant age effect on almost all microglia parameters in every auditory nucleus studied but also a significant effect of hearing on all microglial measurements in the VCN, the percentage of CD68 occupancy of microglia in every auditory nucleus studied, and the soma area and territory area of microglia in the IC. A significant NIHL-by-age interaction was observed only for the percentage of CD68 occupancy in microglia in the VCN (F_4, 61_ =5.774, P<0.001).

**Table 1:** Summary of the significance levels of the effects of hearing (NIHL vs. control) and age (time point) on the parameters of microglia in the auditory pathway determined using two-way ANOVA.

	**Hearing loss**					**Age**			
	**VCN**	**DCN**	**IC**	**AC**		**VCN**	**DCN**	**IC**	**AC**
Microglial density	***	ns	ns	ns		***#***	***#***	***#***	***###***
Percentage of CD68 occupancy	***	**	***	*		***###***	***###***	***###***	***###***
Average soma area	**	ns	*	ns		***#***	ns	ns	***#***
Average territory area	***	ns	*	ns		***###***	***#***	***###***	***###***
Total process length per cell	*	ns	ns	ns		***###***	***###***	***###***	***###***
Total process endpoints per cell	**	ns	ns	ns		***#***	***###***	***###***	***###***

The significance levels for the effects of hearing (NIHL vs. control) and age (time point) are represented by the * and # symbols, respectively: */#: P<0.05; **/##: P<0.01; ***/###: ^#^P<0.001; ns, not significant. VCN, ventral cochlear nucleus; DCN, dorsal cochlear nucleus; IC, inferior colliculus; AC, auditory cortex.

**Figure 5:**
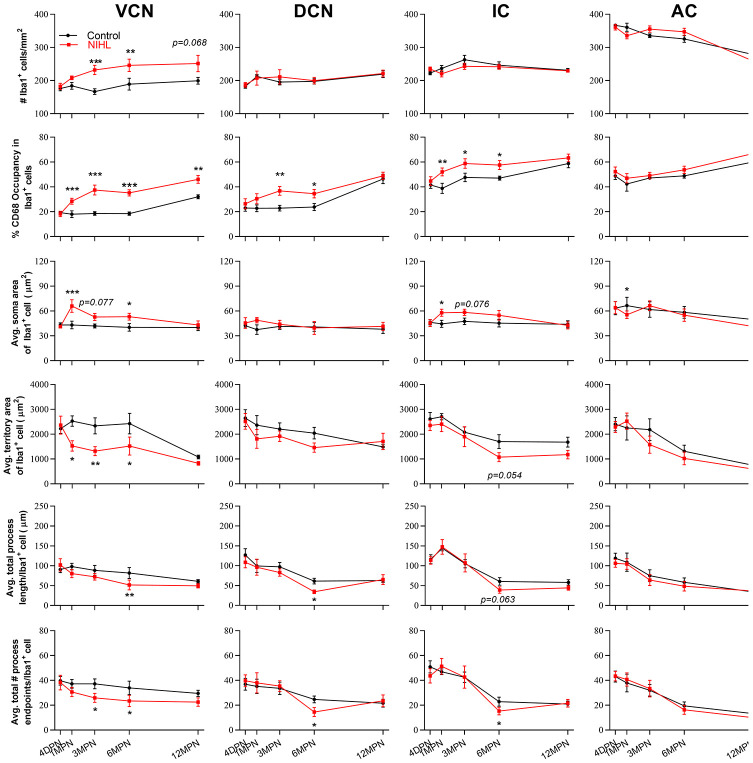
**The impact of NIHL and age on the morphology of microglia in auditory brain regions.** Quantification of the impact of NIHL on each individual parameter of microglia in the VCN, DCN, IC, and AC by post hoc pairwise comparisons between groups as a function of age. The values are presented as the mean ± SEM of 6-8 mice per group. *P<0.05, **P<0.01 and ***P<0.001 in post hoc comparisons between age-matched groups after two-way analysis of variance (ANOVA), showing a significant effect of NIHL.

Post hoc pairwise comparisons (Figure 5, Tukey method) revealed that compared to controls, the microglia in the VCN of the NIHL animals displayed significant increases in cell density at 3 MPN and 6 MPN, CD68 occupancy from 1 MPN to 12 MPN and average soma body size at 1 MPN and 6 MPN and significant decreases in average territory area at 1 MPN, 3 MPN and 6 MPN, total process length per cell at 6 MPN and total process endpoints per cell at 3 MPN and 6 MPN. Significantly higher CD68 occupancy than the control was also shown in the DCN at 3 MPN and 6 MPN and in the IC at 1 MPN, 3 MPN, and 6 MPN in the NIHL group. As CD68 is also expressed by homeostatic microglia and upregulated by immune challenges or increased activities, the phagocytic activity of microglia was also evaluated by assessing CD68-immunopositivity (CD68 immunofluorescence integrated density/Iba1 immunofluorescence integrated density within Iba1+ cells) [48]. Consistent with the results of the CD68 occupation analysis, significant increases in CD68 immunoreactivity were observed in NIHL animals (see Supplementary Figure 1). Taken together, these data demonstrated that after acquired HL, prolonged (up to 6 months after noise exposure) microglial activation occurred not only in the CN, which has been illustrated by previous reports [12, 13, 15, 16], but also in the upper part of the auditory brain region.

### Age-related microglial degeneration in the hippocampus is aggravated in mice with NIHL

**Figure 6:**
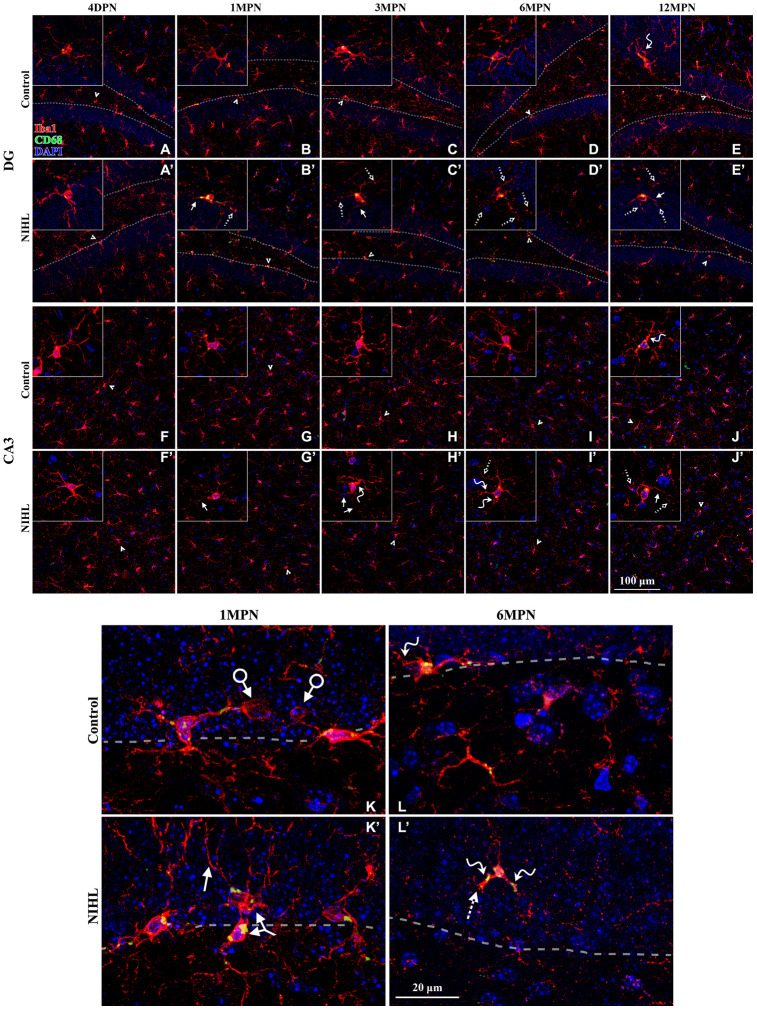
**Microglial deterioration in the hippocampus associated with normal aging is aggravated by NIHL.** (**A**–**J’**) Representative z-projection images of cells labeled with Iba1 (red), CD68 (green), and DAPI (blue) in the DG (**A**–**E’**) and CA3 (**F**–**J’**). Dotted lines in (**A**–**E’**) depict the SGZ (region between the granule cell layer and hilus). Iba1+ cells signified by arrowheads are magnified in the corresponding inserts. Scale bar equals 100 μm. (**K**–**L’**) Representative magnified images of Iba1-labeled cells in the DG from both groups at 1 MPN (**K**–**K’**) and 6 MPN (**L**–**L’**). Dotted lines depict the SGZ. Dystrophic cells are readily distinguished from ramified cells by degenerative changes in their cytoplasmic structure, such as deramified/atrophic cells (solid line arrows in **B’**, **C’**, **E’**, **J’**, **H’**, **J’**, and **K’**), fragmented or unusually tortuous processes (wavy arrows in **E**, **J**, **H’**, **I’**, **L**, and **L’**), and the formation of fragmented or beaded processes (dotted arrows in **B’**, **C’**, **D’**, **E’**, **I’**, **J’**, and **L’**). Arrows with circles in (**K**) point to microglial phagocytic pouches, a special modification of the microglial process involved in removing apoptotic cells from the adult SGZ neurogenic niche [27, 28]. The two-headed arrow in (**K’**) points to two abnormally aggregated microglial cells. Scale bar equals 20 μm.

As shown in Figure 6, Iba1^+^ microglial cells were widely distributed in the DG and CA3 of both control and NIHL mice. In control mice, microglia in young 4 DPN and 1 MPN subjects displayed a highly ramified phenotype with abundant, branched processes and continuous Iba1 distribution (Figure 6A, 6B, 6F–6G). Phagocytic pouches, ball-and-chain structures (connected to the microglial cell body at its terminus) implicated in shaping adult hippocampal neurogenesis by eliminating apoptotic cells in the SGZ under healthy conditions [27, 28], were present in the magnified images (arrows with circles in Figure 6K). Some of the microglia in control 6 MPN (Figure 6D, 6L) and 12 MPN mice (Figure 6E, 6J) showed more senescent/dystrophic features (reduction in soma size, thickening of shorter processes, and deramification of the cells [35, 47]) than young 4 DPN and 1 MPN mice, indicating age-related microglial deterioration (senescence/dystrophy) in the hippocampus [32, 34, 35].

No detectable difference in microglial morphology was observed between control and NIHL mice at 4 DPN (Figure 6A vs. 6A’, 6F vs. 6F’). Compared with age-matched control mice, NIHL mice exhibited enhanced CD68 expression in the DG at 1 MPN and decreased Iba1^+^ cell density in the DG and CA3 at and/or after 1 MPN. Multicellular aggregates were occasionally observed in NIHL mice (two-headed arrow in Figure 6K’). Quantification of the spatial distribution of microglia [44] in the DG showed the significant compromise of the homogeneous distribution of microglial cells in NIHL mice (see Supplementary Figure 2). Furthermore, several morphological hallmarks of microglial dystrophy, such as significant reductions in soma size [35] (Figure 6B’, 6C’, 6G’, 6H’) and thinning and deramification of processes, resulted in a significant reduction in spatial coverage [49, 50] [32] (solid arrows in the insets of Figure 6B’, 6C’, 6E’, 6G’, 6H’, 6J’, and in Figure 6K’), the appearance of thickened and unusually tortuous processes [49, 50] [32] (wavy arrows in the insets of Figure 6H’–6L’), and/or the formation of fragmented or beaded processes with discontinuous and/or punctate Iba1 labeling [34, 50] (dotted arrows in the insets of Figure 6D’, 6E’, 6I’–6L’), occurred earlier and more frequently in NIHL mice than in control mice. Consequently, a larger area of the brain parenchyma was no longer covered by microglial processes in older NIHL mice than in control mice. All these features indicate aggravated microglial deterioration [34, 35, 46, 47, 50] in the hippocampus of NIHL mice.

Two-way ANOVA was performed to evaluate the effect of the factors hearing (NIHL vs. control) and age (time point) on the microglial measures. As summarized in Table 2, significant effects of both age and hearing were observed on almost all parameters. A significant hearing-by-age interaction was observed for average soma area in the DG (F_4, 55_ =3.005, P=0.026) and CA3 (F_4, 54_ =7.085, P<0.001).

**Table 2:** Summary of the significance levels of the effects of hearing and age on the parameters of microglia in the DG and CA3 determined using two-way ANOVA.

	**Hearing loss**			**Age**	
	**DG**	**CA3**		**DG**	**CA3**
Microglial density	**	***		***#***	***##***
Percentage of CD68 occupancy	ns	**		***##***	ns
Average soma area	***	***		***###***	***###***
Average territory area	**	***		***###***	***###***
Total process length per cell	***	ns		***###***	***##***
Total process endpoints per cell	***	ns		***##***	***#***

The significance levels for the effects of hearing (NIHL vs. control) and age (time point) are represented by the numbers of * and # symbols, respectively: */#: P<0.05; **/##: P<0.01; ***/###: P<0.001; ns: not significant.

**Figure 7:**
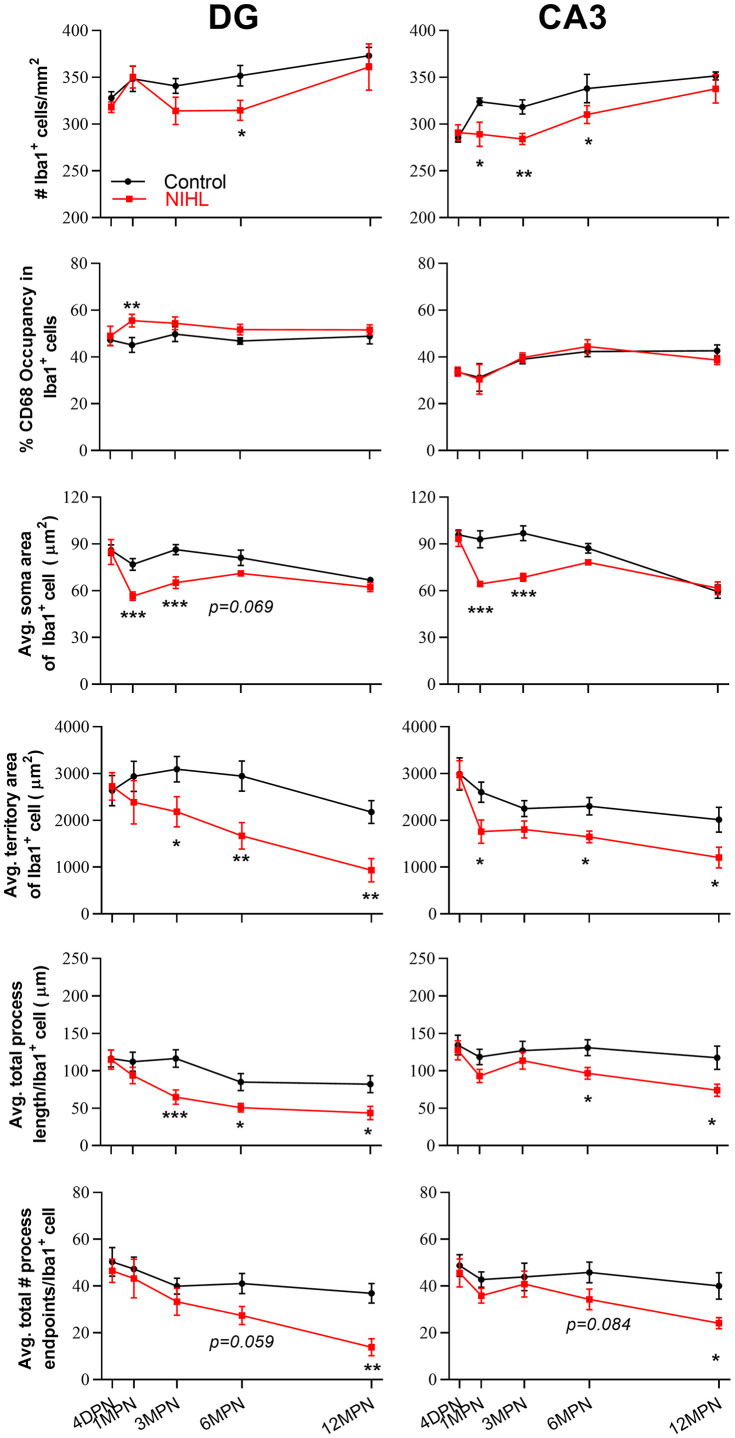
**Quantification of the impact of hearing on microglial parameters in the DG and CA3 by post hoc pairwise comparisons between groups as a function of age.** The values are presented as the mean ± SEM of 5-8 mice per group. *P<0.05, **P<0.01 and ***P<0.001 in post hoc comparisons between age-matched groups after two-way ANOVA, showing a significant effect of NIHL.

Figure 7 shows the effect of hearing on each parameter by post hoc pairwise comparisons between groups (Figure 7, Tukey’s method) as a function of age. A lower density of microglia was observed in the DG of the NIHL group at 6 MPN and in the CA3 at 1, 3 and 6 MPN. Additionally, a transient increase in CD68 occupancy was observed in the DG at 1 MPN (consistent with the quantitation of CD68 immunoreactivity, Supplementary Figure 1), smaller soma areas were observed in both the DG and CA3 at 1 MPN and 3 MPN, a lower territory area and total process length were observed in both the DG and CA3 at 6 MPN and 12 MPN, and fewer total process endpoints were observed in both the DG and CA3 at 12 MPN.. Taken together, these data demonstrated that age-dependent microglial dystrophy is aggravated in NIHL animals.

### Hippocampal neurogenesis is significantly correlated with hippocampal dystrophic microglial morphology

**Figure 8:**
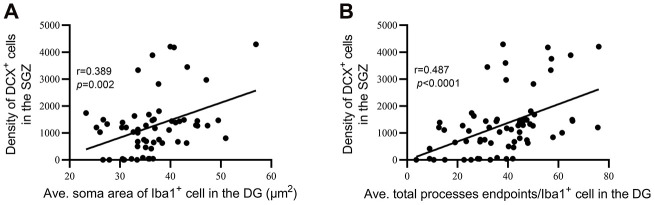
**Hippocampal neurogenesis was significantly correlated with dystrophic microglial morphology.** (**A**) Correlation between the average soma area of Iba1^+^ cells in the DG and the density of DCX^+^ cells in the SGZ. (**B**) Correlation between average total process endpoints per Iba1^+^ cell in the DG and density of DCX+ cells in the SGZ.

To estimate the relationship between hippocampal neurogenesis and microglial dystrophy, we performed correlation analyses between DCX density and the average soma area of microglia and between DCX density and the average total number of microglial process endpoints in the DG of all animals. The analysis revealed a positive correlation between the average soma area of Iba1^+^ cells in the DG and the density of DCX^+^ cells in the SGZ (r=0.389, P=0.002, Figure 8A), as well as a positive correlation between the average total process endpoints per Iba1^+^ cell in the DG and the density of DCX^+^ cells in the SGZ (r=0.487, P<0.0001, Figure 8B).

## DISCUSSION

Because of the rapid urbanization and industrialization of modern society, noise pollution is more severe and more widespread than ever before. Noise-induced hearing loss (NIHL) can be the result of both long-term, repeated exposure to excessive noise and a single exposure to an extremely intense sound that causes damage to the auditory sensory cells in the cochlea. The results from a number of epidemiological studies have demonstrated a significant link between HL and cognitive decline [2, 4, 6, 51–55]. Considering the abundance of opportunities for noise overexposure and the enormous global challenge faced in the prevention of dementia due to its rising prevalence and the lack of an effective cure, it is imperative to explore the relationship between HL and cognitive function as well as the underlying mechanism in animals with NIHL.

The hippocampal DG plays a pivotal role in cognitive function, including the formation of new memories and spatial navigation [56], and is one of the most affected areas in dementia [57–59]. The subgranular zone (SGZ), a narrow band of tissue between the granule cell layer and the hilus of the hippocampal DG, is one of the regions where adult neurogenesis takes place [60]. Newborn granule cells generated in the SGZ grow dendrites into the molecular layer and send their axons into the cornu ammonis (CA3) region to integrate into pre-existing neuronal circuits [60]. Increasing evidence from both experimental and clinical studies indicates that hippocampal neurogenesis is essential for hippocampal-dependent learning and memory [61, 62]. The close association between the decline in neurogenesis and cognitive deterioration during aging, coupled with the disruption of neurogenesis and cognitive dysfunction observed in mouse models of dementia, has led to the general consensus that impairment of neurogenesis is a relevant mechanism underlying cognitive impairments [63–66].

Our previous work demonstrated that NIHL mice exhibited decreased adult neurogenesis in the DG of the hippocampus [8, 9, 67]. This result is, at least in part, consistent with those obtained from an epidemiological study in which hearing-impaired individuals showed significantly smaller hippocampal volumes than their non-hearing-impaired counterparts [68] and thus suggested a causal role of acquired peripheral HL in dementia and the involvement of hippocampal neurogenesis in the underlying mechanism [8, 9, 67]. The present study further examined the influence of NIHL on the age-related decline in neurogenesis in the SGZ and explored the possible mediator of the influence. Considering the prolonged influence of acquired peripheral HL on the microglial phenotype [12, 13, 15, 16] and the central role of microglia in the development of neurodegenerative disease [18, 42, 69], we suspected that microglia might be a precipitating factor between HL and hippocampal neurogenesis. Consistent with this hypothesis, our results show evidence of NIHL-induced microglial pathogenesis at multiple locations along the central auditory pathway as well as the hippocampus, and these changes seem to be significantly correlated with the NIHL-induced decline in hippocampal neurogenesis.

Adult neurogenesis in the hippocampus comprises a series of sequential events, including cell proliferation and survival, cell differentiation and migration, and morphological maturation and functional integration of newly generated neurons into the hippocampal circuitry [70, 71]. It has been well established that adult hippocampal neurogenesis declines with age, and this decline is a major contributing factor to the development of cognitive deficits and dementia [63, 72]. Here, we show that, consistent with the previously reported age-dependent decline in neurogenesis [63, 73], the densities of both Ki67^+^ cells and DCX^+^ cells in the DG decreased in both the NIHL and control groups with age. Compared with the findings in age-matched control mice, the densities of Ki67^+^ cells and DCX^+^ cells were clearly reduced in NIHL mice at 1 MPN and 3 MPN, while no significant differences between age-matched groups were shown at 4 DPN and after 6 MPN, suggesting that NIHL accelerates the age-related decline in neurogenesis. From 1 MPN to 6 MPN, DCX^+^ cells in the NIHL mice exhibited smaller and more irregularly shaped somas and shorter neurites than cells in the age-matched control mice. The Sholl analysis revealed a significant decrease in the complexity of DCX^+^ cells in the DG of NIHL mice at 1 MPN (see Supplementary Figure 3), providing further morphological evidence for the disruption of neurogenesis in NIHL mice.

The NIHL model was established in this study at young adulthood, which matched the onset of NIHL in humans at least in industrialized countries. The significant effect of hearing on hippocampal neurogenesis was only observed at 1 MPN and 3 MPN (4 and 6 months of age) in the present study. Thus, the maximal hippocampal impairment associated with acquired HL is observed soon after the onset of HL, suggesting a time window at which the early intervention may protect these individuals from further neurodegenerative changes. We are currently unable to provide a clear explanation for why the density of Ki67^+^ cells and DCX^+^ cells was approximately the same after 6 MPN between the NIHL and control groups; however, at least two explanations are plausible. First, the effect of aging on the proliferation in hippocampus is more dominant than the effect of NIHL at older ages. This hypothesis was supported by the substantial decrease in the density of Ki67^+^ cells or DCX^+^ cells after 6 MPN (Figure 2). Therefore, a floor effect might have masked the effects of NIHL established at young age on hippocampal neurogenesis at older ages. Second, the post-lesion neuroplasticity may partially compensate for the effect of sensory deprivation if it is established at young age [74]. Based on these findings, we are unable to completely exclude the possibility that the findings observed in subjects suffering from acquired HL might differ if the age of HL onset is older. In humans, the prevalence of acquired sensorineural HL increases dramatically in elderly individuals and generally occurs in a short period beginning at 60 years of age. Older mice, particularly in mice in which long-term NIHL was established, but not a loud noise presented for several hours, should be analyzed in future studies.

The process of adult hippocampal neurogenesis is highly regulated by a variety of microenvironmental factors in the neurogenic niche [30, 61]. Increasing evidence indicates that ramified microglia are an essential component of the neurogenic niche in the SGZ and play a critical supporting role in hippocampal neurogenesis by eliminating apoptotic newborn cells and secreting trophic factors that can influence the proliferation, differentiation, and survival of newborn cells [26–28, 30]. Microglial phagocytosis of apoptotic debris plays a critical role in maintenance of the neurogenic cascade by preventing primary apoptotic newborn cells from undergoing secondary necrosis, which may lead to an inflammatory response and thus be detrimental for adult neurogenesis [27, 36, 75]. Furthermore, microglia sense and respond to signals from the surrounding environment by secreting inflammatory cytokines and neurotrophic factors that regulate hippocampal neurogenesis in a temporally and spatially specific manner [30, 76].

It has been well established that microglial morphology is inextricably linked to their functions [20, 42, 49, 77]. In the healthy CNS, microglia have a highly ramified morphology with a small soma and abundant, long, thin, and branched processes, which allow them to continuously screen the surrounding parenchyma for injuries or pathogens [20, 49]. In this state, microglia were previously described as “resting.” However, more recent studies demonstrate that even in the normal brain, microglia are highly dynamic and constantly survey their territorial domains and protect neurons from homeostatic irregularities [20, 77–79]. Therefore, the name "resting" microglia is slowly being replaced with “ramified/surveillant” microglia [77, 80]. Ramified microglia can respond to disturbance or loss of brain homeostasis (such as trauma, neurodegenerative diseases, or altered neuronal activity), indicating real or potential danger to the CNS, with profound changes in cell shape and functional behavior, which is summarily defined as “microglial activation” [20]. Activated microglia are usually characterized by a larger soma, shorter and thicker processes, decreased ramification, and increased expression of activation markers such as CD68, a glycoprotein that localizes to lysosomes [20, 81]. Microglial activation cannot be simply categorized as harmful or beneficial [82]. Regulated activation and proper termination might help in tissue preservation, repair, and renewal, while intense acute or chronic activation may result in irreversible neurotoxicity and neurodegeneration [20, 21, 80, 83]. Therefore, microglia perform their functions at optimal levels are necessary for the maintenance of homeostatic functions and overall health. Degenerative microglia, characterized by dystrophic morphological features, including deramification (distinct loss of fine branches) and shortening, twisting and fragmentation of their processes, have been recently reported in the aged and AD hippocampus [32, 34, 49, 84–87]. W.J. Streit first introduced the concept of “dystrophic microglia” based on observations of microglial morphology in the human brain [34, 43, 87–91]. Dystrophic microglial cells are believed to lose their neuroprotective functions [34, 92], thereby driving the progression of age-related neurodegenerative pathologies, such as AD [32, 34, 47, 49, 50, 87, 92, 93, 17, 89]. The replacement of microglia in the aged brain with new microglia results in increased hippocampal neurogenesis and improved cognition in aged mice [94], providing further evidence for the contribution of microglial degeneration to age-related cognitive impairments and increased susceptibility to neurodegenerative disorders, including dementia [18, 32, 34, 35, 69, 84, 86, 87, 89–91, 95].

Qualitative assessment of cell morphology is a reliable way of distinguishing among ramified, activated, and dystrophic microglia [34]. A recent study performed using postmortem human hippocampal tissues revealed marked microglial degeneration in patients with AD, which was particularly evident in the DG and CA3 subfields [85]. The DG-CA3 region plays a key role in sensory (including auditory) information processing by the hippocampus [40] and was reported to be highly sensitive to changes in sensory input [96]. Thus, we focused our attention on microglial morphology in the DG and CA3. In line with previous reports [34, 97], we observed age-related changes in microglial morphology, including shrinkage of the cell soma and territory area and decreased cell complexity. Compared to microglia in aged-matched controls, microglia in NIHL mice display significant reductions in cell density, soma area, territory area, process length and process endpoints, with the exception of 4 DPN. Furthermore, at 6 MPN and 12 MPN, fragmented microglia (the principal feature of microglial dystrophy [32, 34, 47, 98]) lacking an intact morphology were observed more frequently in NIHL brains than in control brains. All these data suggest that aggravated age-associated microglial degeneration occurs in NIHL mice.

The major functions of ramified microglial cells are to constantly interact with different CNS components and to play a central role in the maintenance of brain homeostasis [87]. Wear and tear on microglia could be crucial for the development of neurodegenerative disease through diminution of neuroprotective functions, direct increases in neurotoxicity, and dysregulated responses to signals and perturbations [47]. In this study, dystrophic microglia were observed much earlier and much more frequently in the DG of NIHL mice than in control mice. The reductions in microglia density and coverage area per cell in NIHL animals imply an overall deficiency of essential functions performed by microglia. Correlation analysis between parameters of hippocampal neurogenesis (DCX^+^ cell density) and hallmarks of microglial dystrophy (average soma size and average total number of process endpoints of microglia) in the DG revealed that the decline in hippocampal neurogenesis was significantly correlated with dystrophy of microglia in the DG. Therefore, considering the essential contribution of microglia to adult hippocampal neurogenesis [26–28, 30] as well as the contribution of microglial dystrophy to the onset of neurodegeneration [34, 82, 87, 88, 98], we consider that hippocampal microglial dysfunction (as evidenced by dystrophic morphology) might contribute to acquired hearing loss-related hippocampal neurogenesis impairment by exacerbating aging-related cognitive decline.

Microglial function is tightly regulated by factors within the CNS microenvironment. Increasing evidence suggests that disturbances in the brain environment, such as neurotrophic cytokines and inflammatory factors, can have profound consequences for microglial phenotype and function [99]. Several animal studies have reported that damage to the inner ear, which reduces neural output from the cochlea, induces long-term microglial activation in the cochlear nucleus [12, 13, 15]. The present study demonstrated that compared with microglia in the age-matched control group, microglia in the VCN, DCN and IC of the NIHL group, to various degrees, assumed an activated morphology at 1 MPN, 3 MPN, 6 MPN, and 12 MPN, as evidenced by increased CD68 immunoreactivity, increased soma area, reduced territory area and decreased cell complexity (represented by average total process length and endpoints per cell). Meanwhile, no significant difference in microglial morphology was observed in the visual cortex between age-matched groups (see Supplementary Figure 4). These data extended previous findings and provided broader insight into underlying cellular events that occur in multi-auditory brain regions after acquired HL. The brain is a finely tuned machine that has an amazing capacity to continually undergo physiological changes in response to environmental stimuli. Neural circuits that are not actively engaged in task performance for an extended period of time begin to degrade [100]. This “use-it-or-lose-it” principle also applies to the auditory system [14, 51]. Numerous studies have suggested that the interruption of neuron-microglia communication plays an important role in regulating the microglial phenotype [99, 101] and that microglia play a fundamental role in monitoring neuronal activity, surveilling the neuronal milieu, facilitating structural remodeling of neurons and modulating neuronal and synaptic plasticity [39, 69]. Thus, a plausible speculation is that microglia in the auditory region of the brain are activated by disruption of the microglia–neuron communication (such as a fractalkine signaling deficiency [102]) and that the activated microglia in the auditory region of the brain may play a pivotal role in remodeling during the process of circuit plasticity following acquired HL, including activity-dependent synaptogenesis and synapse elimination [13, 16, 103–105].

The hippocampus is believed to be involved in auditory perception [40, 106–110]. Therefore, another reasonable hypothesis is that acquired peripheral HL might alter the status of microglia in the hippocampus at least partially through mechanisms related to neuronal activity. However, the microglia in the hippocampus of NIHL mice did not show as many activated morphological characteristics as those in the auditory brain but instead showed more dystrophic features. We currently do not have an explanation for this discrepancy other than the brain region-dependent diversity of microglia that is thought to enable regionally localized homeostatic functions and underlie region-specific sensitivities to microglial dysregulation and regional variation in susceptibility to age-related neurodegeneration [111]. Hippocampal microglia were reported to maintain a more immune-alert state and are particularly vulnerable to the environment during aging [111, 112]. The site-specific vulnerability of the hippocampus to internal and external changes as well as to AD pathology is supposed to be related in part to the specific vulnerability of hippocampal microglia [112–114]. Kreisel, T. et al. demonstrated that chronic stress produces microglial dystrophy in the hippocampus but not in other brain regions [35], providing further evidence for the region-specific response of hippocampal microglia to stimuli. Several possibilities should be considered in future studies examining the region-dependent reaction of microglia to aging and HL, including the differences in the neural connectivity, activities during neurogenesis, and the density of microglia between the auditory region of the brain and hippocampus. On the other hand, noise exposure has been associated with the stress response, and the hippocampus is highly susceptible to stress. Based on accumulating evidence, stress exerts profound effects on microglia [115]. Although in our previous study, only a transient increase in the levels of stress hormones was observed in CBA mice after a noise exposure similar to the noise used in the present study [9], the possible contribution of stress to the degenerative process observed in NIHL mice should be further investigated in future studies.

In summary, we found the age-related decline in hippocampal neurogenesis was accelerated in mice with NIHL and observed NIHL-related morphological alterations in microglial cells, including morphological changes implying activation in the auditory pathway and dystrophy in the hippocampus. In addition to previous reports describing increased inflammatory responses in the auditory brain regions following HL, our data provides the first evidence that accelerated microglial degeneration in the hippocampus may contribute to the HL-related impairment in neurogenesis. Considering the hypothesis of a causal relationship between microglial degeneration and the onset of neurodegeneration, i.e., neurodegeneration may be secondary to microglial damage [34, 89–91], the findings of the present study are appealing because they take into account the idea that acquired HL contributes to the pathogenesis of sporadic AD by exacerbating aging-related degeneration of hippocampal microglia. These findings also provide further explanation as to why anti-inflammatory drugs are ineffective at preventing or diminishing neurodegeneration and dementia [34]. Exactly how the deterioration of hippocampal microglia is linked to NIHL has not yet been determined, but this may become a fertile area of investigation in future research.

## MATERIALS AND METHODS

### Animals

Eighty adult (6-7 weeks old) male CBA mice (Shanghai Super B&K Laboratory Animal Corp. Ltd., Certificate No. SCXK(HU) 2013-0016) were housed, four per cage, under standard conditions (8 a.m. to 8 p.m. light cycle, 22 °C, 55% humidity, and ad libitum access to food and water). All procedures were approved by the University Committee for Laboratory Animals of Southeast University, China (SCXK2011-0003). All mice were screened for a normal Preyer reflex to rule out HL prior to the start of the study. The mice were randomly assigned to a control group (n=40) and an NIHL group (n=40). Mice in the NIHL group were exposed to broadband noise for 2 h at an SPL of 123 dB. The mice in the NIHL group were further divided into five subgroups (n=8/group) and sacrificed for neuroanatomical analysis at 4 days post-noise exposure (4 DPN) and 1, 3, 6, or 12 months post-noise exposure (MPN). The mice in the control group were subdivided into five groups (n=8/group) and sacrificed at times matching those in the five NIHL groups.

### Noise exposure

Noise exposure was carried out as described previously [8, 9]. Briefly, after a 30-min acclimation to the noise-exposure environment, conscious and unrestrained mice were placed individually in metal wire mesh cages (cage size: 11 cm×11 cm×23 cm) located 40 cm below the horns of two loudspeakers (an NJ speaker YD380-8bH and a BM professional speaker HG10044XT, nominal bandwidth 1-20 kHz). The noise signal was generated by a System III processor from Tucker-Davis-Technologies (TDT, Alachua, FL, USA) and amplified by a Yamaha P9500S power amplifier. The noise level was calibrated to an SPL of 123 dB and monitored during the exposure using a 1/4-inch microphone linked to a sound level meter (Larson Davis, Depew, NY, USA; microphone: 2520, sound level meter: 824, from Larson Davis, Depew, NY, USA). The variation in sound level across the cage was less than 1 dB. Control mice were subjected to an identical procedure except the noise was not turned on (sham exposure).

### Auditory brainstem response (ABR) assessment

ABRs were recorded for all mice (except the 4 DPN subgroups) in the NIHL and control groups at 3 weeks post-exposure. The ABR was assessed in a sound-attenuating chamber. Mice were anesthetized by intraperitoneal injection of ketamine plus xylazine (40 mg/kg + 10 mg/kg, respectively). TDT hardware (RZ6, System III) and software (BioSig and SigGen) were used for signal generation and ABR acquisition. The acoustic stimuli were tone bursts of a 10-ms duration with cos^2^ gated, 0.5-ms rise/fall times. The tone bursts were played out through an MF1 speaker located 10 cm in front of the animal’s head. Stimuli were presented at a rate of 21.1/s at frequencies of 2, 4, 8, 16 and 32 kHz. Three subdermal needles were used as electrodes for ABR recording (noninverting electrode at the vertex, inverting electrode beneath pinna and ground electrode at the hind limb). The evoked responses were sampled at 25 kHz, amplified by an RA16PA preamplifier (TDT) with a gain of 20, and averaged 1000 times. At each frequency, testing began at an SPL of 90 dB, and the level was decreased in 5-dB steps until the ABR response disappeared. The ABR threshold at each frequency was defined as the lowest intensity that yielded a detectable peak III ABR response. Throughout the recording and recovery from anesthesia, the animal’s body temperature was maintained at 37.5 °C using a thermostatic heating pad. Since the maximal level of the signal was an SPL of 90 dB, the threshold was defined as an SPL of 95 dB if the ABR was not detectable at an SPL of 90 dB.

### Immunohistochemistry

At a defined time of sacrifice, mice were deeply anesthetized with pentobarbital (100 mg/kg, i.p.) and transcardially perfused with 0.9% saline followed by 4% paraformaldehyde in 0.1 M PBS. The brains were quickly removed, postfixed (4% paraformaldehyde for 6-8 h at 4 °C), cryoprotected (30% sucrose, until the brain sank), embedded (OCT, Leica), snap-frozen, and then stored at -80 °C. Serial coronal sections (40-μm-thick) of the whole brain were obtained and stored in cryoprotectant solution (30% ethylene glycol, and 25% glycerin in 0.1 M phosphate buffer) at –20 °C. Across the hippocampus, six sections (320 μm apart) per animal were included for the study of neurogenesis or microglia. For the ventral cochlear nucleus (VCN), dorsal cochlear nucleus (DCN), inferior colliculus (IC), and auditory cortex (AC), three coronal sections per region of interest (sections 120 μm apart for the VCN and DCN, sections 320 μm apart for the IC and AC) per animal were used to study microglia.

Staining was performed using selected free-floating sections. A 1-h block with blocking solution was followed by incubation with the following primary antibodies overnight at 4 °C: rabbit anti-Ki67 (for proliferating cells; Abcam, 1:250, ab66155), rabbit anti-DCX (doublecortin, for newly generated neurons; Abcam, 1:1000, ab18723), guinea pig anti-DCX (Millipore, 1:1000, AB2253), rabbit anti-Iba1 (ionized calcium binding adaptor molecule 1, for microglia; Wako, 1:1000, 019-741), and rat anti-CD68 (cluster of differentiation 68, for phagocytic microglia; BIO-RAD, 1:1000, MCA1957). After extensive washing, the sections were incubated with the appropriate secondary antibody for 2 h at room temperature: Alexa-568 goat anti-rabbit (Abcam, 1:1000, ab175471), Alexa-488 goat anti-rabbit (Abcam, 1:1000, ab150077), Alexa-488 goat anti-rat (Thermo Fisher Scientific, 1:1000, A11006), and Alexa-568 goat anti-guinea pig (Abcam, 1:1000, ab175714). All sections were counterstained with DAPI (Beyotime, 1:600, C1027) to visualize cell nuclei. The sections were then mounted on gelatinized glass slides and coverslipped using Antifade Mounting Medium (Beyotime, P0126). To ensure that the observed immunoreactivity was not non-specific staining, random negative control sections were processed in the same manner, with either the primary or secondary antibody omitted.

### Image analysis

Images were captured using a fluorescence microscope (OLYMPUS BX53, Japan) or a confocal microscope (Olympus FV1000, Japan). Samples were analyzed by an observer blinded to the experimental treatment using ImageJ software (US National Institutes of Health, Bethesda, MD, USA). Maximum intensity projections of confocal z-series stacks (at least 25 optical sections at an interval of 1 μm) were created and aligned in the x–y plane to create 2-dimensional images.

Brain regions of interest were confirmed under the microscope by comparing the sections to images in the Allen Mouse Brain Atlas (Allen Institute for Brain Science, 2008). The number of DCX- or Ki67-positive cells in each image acquired through a 20× objective lens was manually counted using the cell counter function of ImageJ in an area of the subgranular zone (SGZ), defined as the SGZ length in the image multiplied by an SGZ height of 20 μm (a layer of cells expanding 5 μm into the hilus and 15 μm into the granular cell layer (GCL) [27]).

Although Iba1 is expressed by peripheral macrophages, it is expressed at high levels in microglia. Microglial cells are easily identified by immunofluorescence staining using Iba1-specific antibodies, enabling an analysis of the morphology of individual microglial cells [35, 116, 117]. Six parameters were employed for the quantitative evaluation of microglial cells in the brain region of interest: (1) microglial density, defined as the number of Iba1^+^ cells per brain area, (2) percentage of CD68 occupancy in microglia, defined as the proportion of CD68^+^/Iba1^+^ cells among all Iba1^+^ cells, which reflects the percentage of phagocytic microglia, (3) average area of microglial soma in an Iba1^+^ cell, (4) average territory area per microglia, defined as the 2-dimensional area outlined by the outermost points of an individual Iba1^+^ cell’s dendritic processes, (5) average total process length per microglia, defined as the average sum of all the lengths of individual Iba1^+^ cell processes, and (6) average total number of process endpoints per microglia, a representation of ramification and complexity.

For all captured images, only cells with a clearly visible cell body were analyzed. The number of Iba1^+^ or Iba1^+^/CD68^+^ cells in each image of brain regions of interest (VCN, DCN, IC, AC, dentate gyrus (DG), and CA3) was manually counted and divided by the counting area to yield the microglial density and percentage of CD68 occupancy in microglia. Microglial soma area was assessed by thresholding and then quantifying Iba1-positive soma areas [35, 118, 119]. The threshold parameters for ImageJ analysis were identical for all examined images. The territory area of microglia (the area encompassed by individual microglia) was measured by manually tracing the contour of the cell, including the tips of dendritic processes, using the smooth polygon tool in ImageJ [33, 116, 118, 119]. The image of a microglia acquired using a z-step interval of 1 μm was binarized and skeletonized using the “threshold” and ‘‘skeletonize’’ functions in ImageJ [116–118]. The total process length and the total process endpoints of an individual microglia were calculated from the skeletonized image with the Skeleton Analysis plug-in bundled in ImageJ [32, 116, 117]. For analysis, only branches longer than 1 μm were counted.

### Statistical analysis

The data were analyzed using SigmaPlot12.0 for Windows (Systat Software Inc) and GraphPad Prism 5 (GraphPad Software). All values are expressed as the mean ± standard error (SE). The level of statistical significance was determined using one- or two-way analysis of variance (ANOVA), followed by *post hoc* Tukey’s test or Student’s two-tailed t-test as appropriate. Data with a non-normal distribution were transformed (using the log rank or Chi-squared test as appropriate) before the analyses to satisfy the prerequisite assumptions of normality. Significance was set at P<0.05.

## Supplementary Material

Supplementary Figres
