# Supplementary material for: Accelerated age-related decline in hippocampal neurogenesis in mice with noise-induced hearing loss is associated with hippocampal microglial degeneration
Source: Aging (Albany NY). 2020 Oct 11;12(19):19493–519. doi: 10.18632/aging.103898 (PMC7732316; doi:10.18632/aging.103898)
Supplement: Supplementary Figres [file aging-12-103898-s001..pdf]

## SUPPLEMENTARY FIGURES

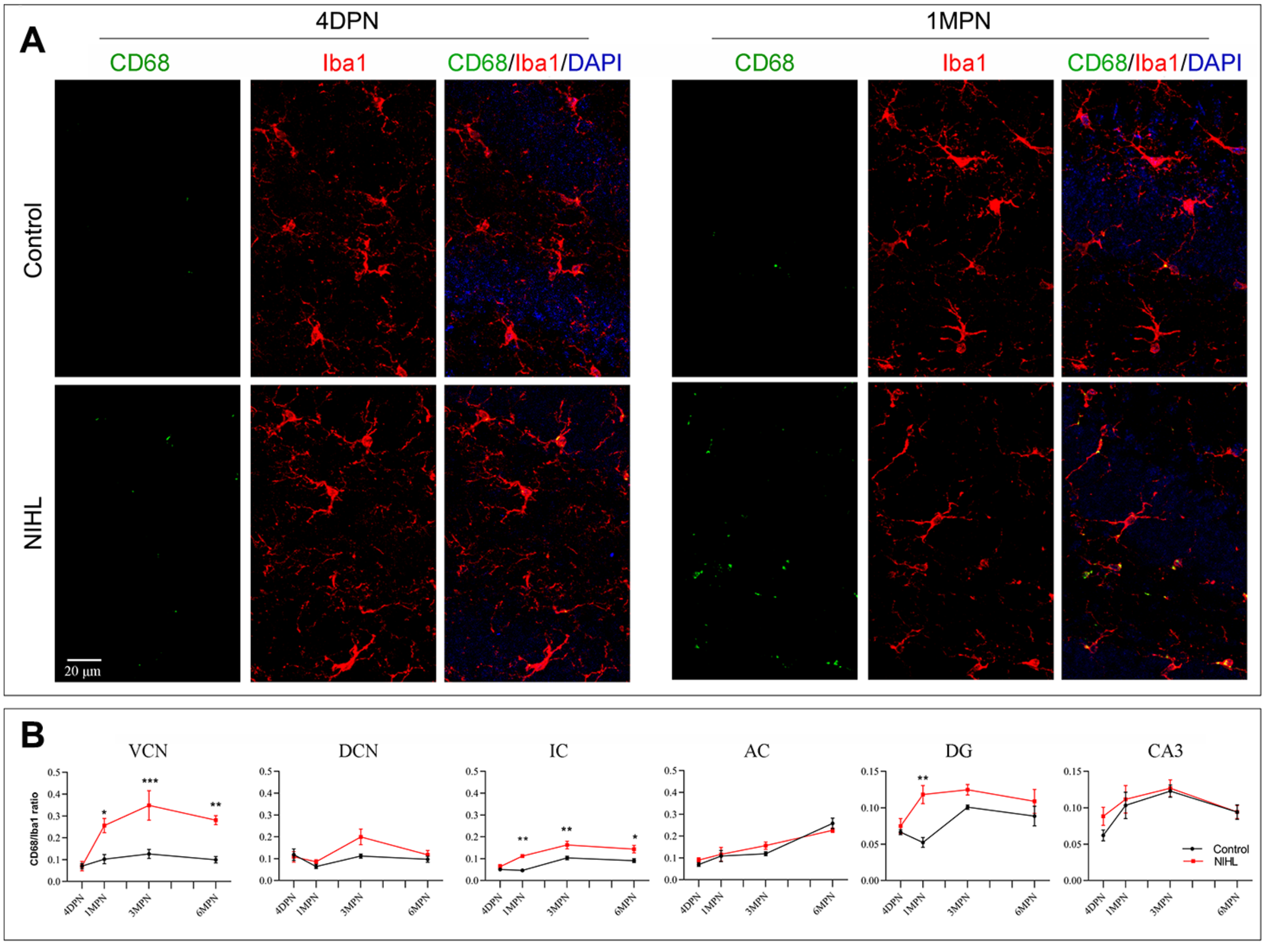


**Supplementary Figure 1. Microglia in the auditory brain regions and hippocampus of NIHL mice exhibited increased CD68-immunopositivity.** (**A**) Representative z-projection images of Iba1-, CD68-, and DAPI-labeled sections of the DG from both groups at 4 DPN and 1 MPN. The scale bar represents 20 µm. (**B**) Quantification of the effect of hearing on CD68-immunopositivity (CD68 immunofluorescence integrated density/Iba1 immunofluorescence integrated density) of microglia in the VCN, DCN, IC, AC, DG, and CA3 by performing post hoc pairwise comparisons between groups as a function of age. The values are presented as the means ± SEM of 5-8 mice per group. *P<0.05, **P<0.01 and ***P<0.001 in post hoc comparisons between age-matched groups after two-way ANOVA, showing a significant effect of NIHL.

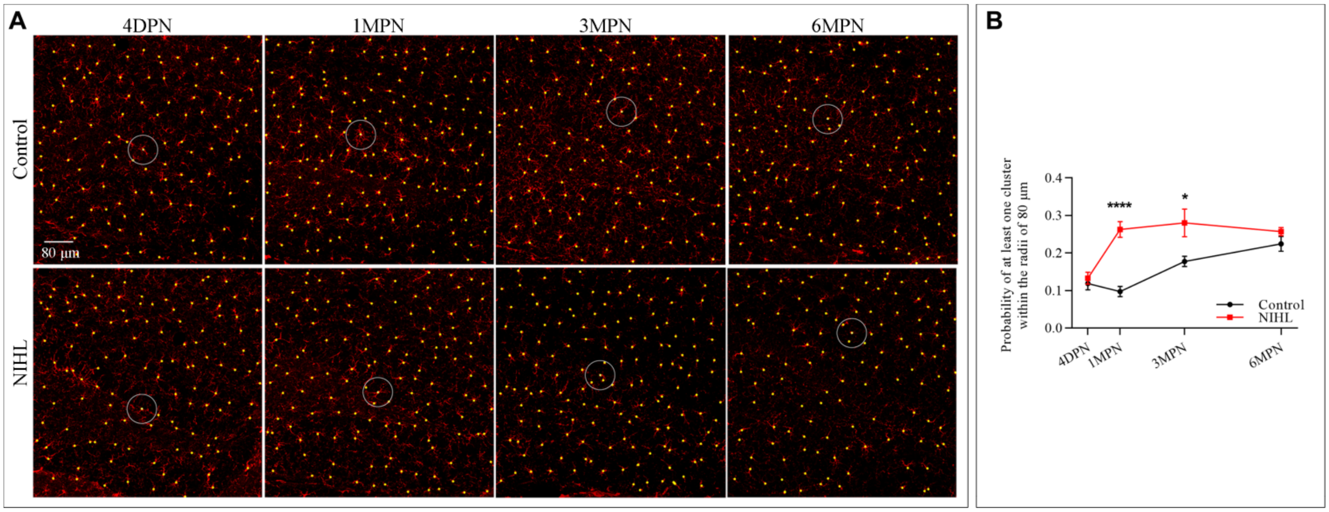


**Supplementary Figure 2. The effects of NIHL and age on the spatial distribution of microglia in the DG.** The numbers of microglia clusters containing three or more cells within a circle with a radius of 80 μm around individual microglia was counted and the spatial distribution of microglia in the DG was quantified by calculating the probability of microglial clusters within the circle (as described previously by Hefendehl et al [1]). (**A**) Representative z-projection images of Iba-1-immunostained sections of the DG from both groups at 4 DPN, 1 MPN, 3 MPN and 6 MPN. The scale bar represents 80 μm. Yellow circles show examples of each microglia used to count neighboring cells. (**B**) Quantification of the effect of hearing on the microglial distribution in the DG by performing post hoc pairwise comparisons between groups as a function of age. The values are presented as the means ± SEM of 5-8 mice per group. A two-way ANOVA revealed significant effects of both age ($F_{3, 39}$ =12.77, P<0.0001) and hearing ($F_{1, 39}$ =29.77, P<0.0001) on the probability of microglial clusters within the circle with a radius of 80 μm in the DG. Post hoc analyses revealed significant effects of hearing at 1 MPN and 3 MPN, and the homogeneous distribution of microglial cells was significantly compromised in NIHL mice (*P<0.05, **P<0.01 and ***P<0.001).

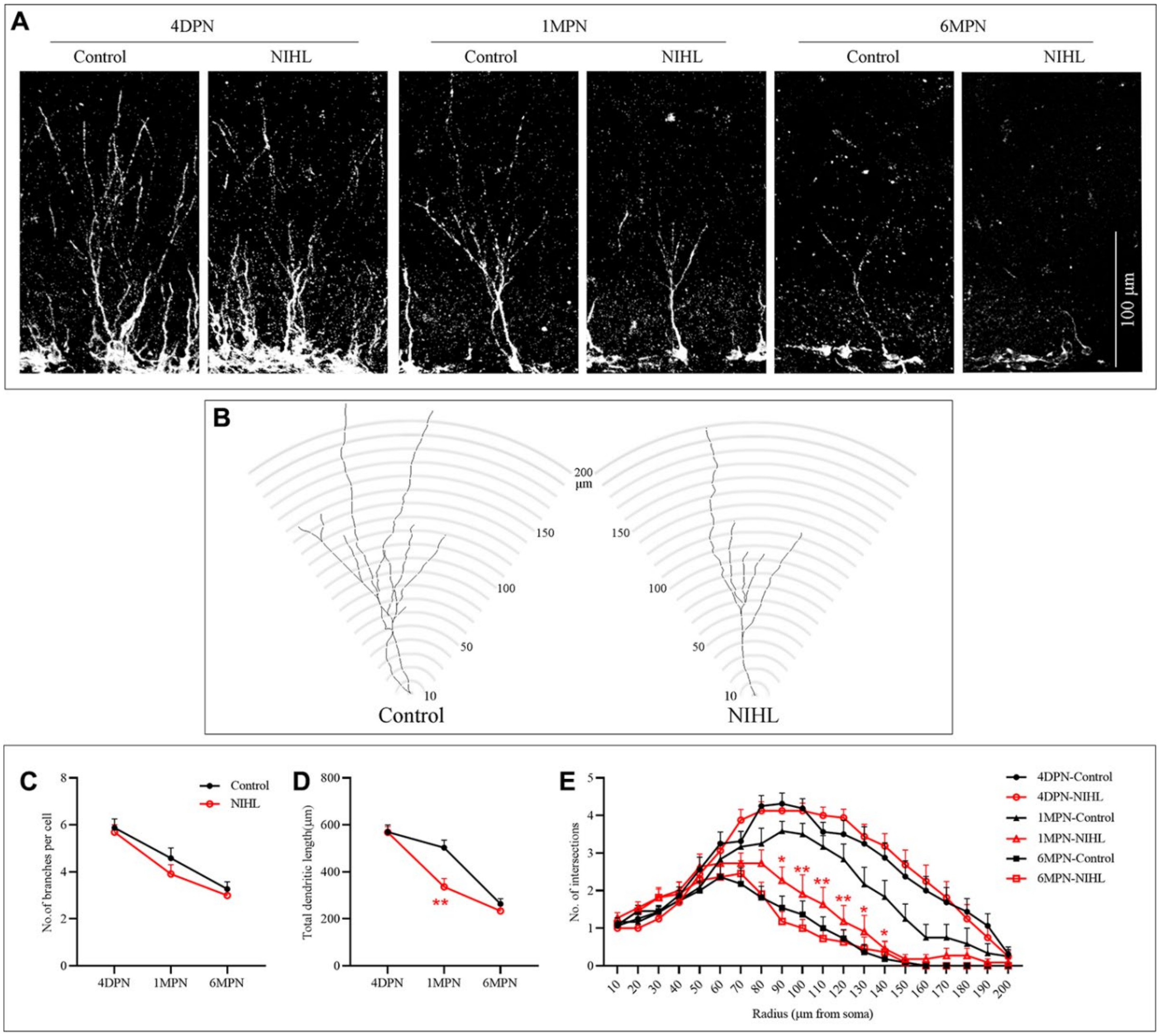


**Supplementary Figure 3. DCX⁺ cells in the DG exhibited significantly decreased complexity in NIHL mice compared to age-matched control mice at 1 MPN.** The morphology of DCX⁺ cells in the DG from both groups at 4 DPN, 1 MPN, and 6 MPN was assessed using a Sholl analysis, which quantifies neuronal complexity by counting the number of neurites that intersect concentric circles drawn at specified intervals from the center of the soma. (**A**) Representative images of DCX⁺ cells in the DG of both groups at 4 DPN, 1 MPN, and 6 MPN. The scale bar represents 100 μm. (**B**) Concentric circles of the Sholl analysis (with increasing radii of 10 μm, ranging from 10 μm to 200 μm from the center of the cell soma) superimposed on the drawing of a DCX⁺ cell in the DG of both groups at 1 MPN. (**C**–**E**) The dendritic complexity of DCX+ cells measured as the total number of branches per cell (**C**), total dendrite length per cell (**D**), and number of intersections in relation to the distance from the soma (**E**). The values are presented as the means ± standard errors (SE) of 8 mice per group (15-20 DCX⁺ cells per mouse). Data for the total number of branches per cell (**C**) and the total dendrite length per cell (**D**) were evaluated using two-way ANOVA (factors: group × age). Numbers of intersections in relation to the distance from the soma (**E**) were evaluated using three-way ANOVA (factors: group × age × radius). *P<0.05, **P<0.01, and ***P<0.001 (Tukey's post hoc test, compared with the age-matched control group).

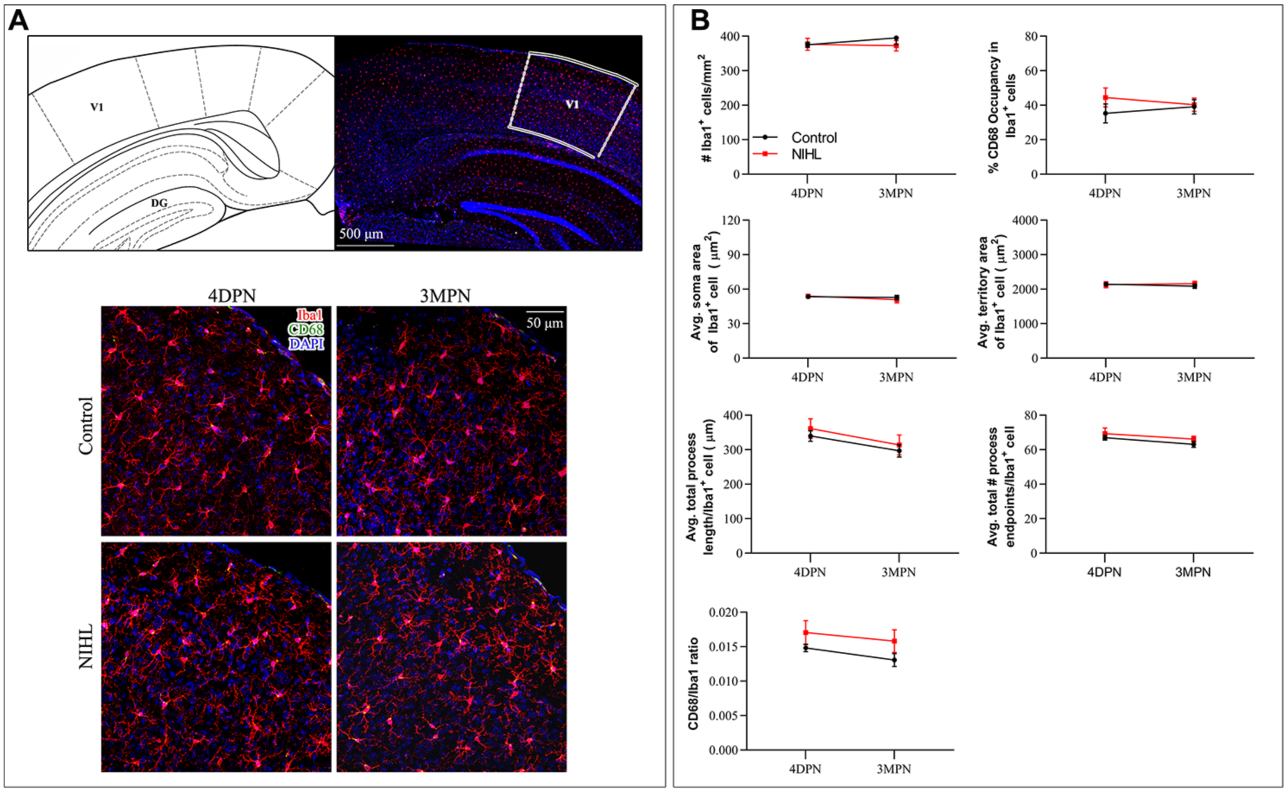


**Supplementary Figure 4. The morphology of microglia in the visual cortex was comparable between the NIHL mice and age-matched controls.** (**A**) Representative z-projection images of Iba1-, CD68-, and DAPI-labeled microglia in the visual cortex of both groups at 4 DPN and 3 MPN. (**B**) Quantification of the effect of hearing on microglial parameters in the visual cortex by performing post hoc pairwise comparisons between groups as a function of age. A significant effect of HL was not observed.

## SUPPLEMENTARY REFERENCE

1. Hefendehl JK, Neher JJ, Sühs RB, Kohsaka S, Skodras A, Jucker M. Homeostatic and injury-induced microglia behavior in the aging brain. Aging Cell. 2014; 13:60–9. https://doi.org/10.1111/acel.12149 PMID:23953759
